# Understanding the Influence of Substance Use, Mental Health, and Stigma on Smoking Behavior Among People Living With HIV: Rapid Qualitative Analysis

**DOI:** 10.2196/97427

**Published:** 2026-10-15

**Authors:** Narayani Ballambat, Dylan R Rice, Reem Najjar, Jessica G Wijesundara, Celine Larkin, Andrew W Liu, Yvonne P Nguyen, Hai Tran, Thi Thanh Xuan Nguyen, Anh Ngoc Do, Nguyen Vu, Melissa Goulding, Jeroan J Allison, Hoa L Nguyen, Rajani Shankar Sadasivam

**Affiliations:** 1 Department of Population and Quantitative Health Sciences, University of Massachusetts Chan Medical School, 55 North Lake Avenue, Worcester, MA, 01655, United States, 1 5088568999; 2 University of Massachusetts Chan Medical School, Worcester, MA, United States; 3 Institute of Population, Health and Development, Hanoi, Vietnam

**Keywords:** HIV, smoking cessation, qualitative research, Vietnam, tobacco use, mobile health, mHealth

## Abstract

**Background:**

In Vietnam, an upper-middle-income country, the majority of people living with HIV smoke. Vietnam has established quitline services to support this population, but they remain underused. Text messaging is widely used in Vietnam, and studies in other countries have shown that text messaging interventions can promote smoking cessation. Our goal was to conduct a formative study to inform the development of a text messaging intervention to increase access to quitline services and promote smoking cessation among people living with HIV who smoke in Vietnam.

**Objective:**

This study aimed to identify factors influencing smoking behaviors among people living with HIV in Vietnam through qualitative interviews to inform a tailored text messaging intervention.

**Methods:**

In October 2022, study staff conducted 30- to 45-minute qualitative interviews with 20 people living with HIV from clinics in Hai Phong and Quang Ninh provinces, Vietnam. Interviews explored factors that influenced smoking, such as substance use, mental health, HIV stigma, social context, and health. A rapid qualitative analysis approach was used to synthesize themes from the data, including identifying key domains based on prior literature, developing a semistructured interview guide, and organizing data within domains to support iterative theme development. These interviews were used to develop prompts to guide people living with HIV who smoke in writing peer messages for a text messaging intervention.

**Results:**

Across 6 themes, participants described smoking as part of their everyday lives, shaped by social situations, stress, alcohol use, and their experiences living with HIV. Social smoking and early initiation were important influences on smoking behaviors, whereas financial concerns and protecting family members and others from secondhand smoke promoted cessation. Some participants used smoking to cope, whereas others wished to quit for better health. HIV stigma was a major contributor to smoking, with many participants describing increased smoking after diagnosis. Participants suggested that cessation support should be better tailored to their needs.

**Conclusions:**

Smoking behaviors among people living with HIV are shaped by overlapping influences, including societal norms, mental health, HIV-related stigma, and other structural stressors. An understanding of these overlapping factors can inform the development of text-based interventions that offer effective cessation support tailored to this population.

## Introduction

Tobacco use contributes to 8 million deaths globally each year, with 7 million attributed to personal tobacco use [1]. The majority of tobacco users live in low- and middle-income countries, and smoking is especially prevalent among people living with HIV [1]. The intersection of smoking and HIV poses a serious public health threat and contributes to disproportionate morbidity and mortality among people living with HIV [2,3]. In Vietnam, an upper-middle-income country, smoking rates among people living with HIV significantly exceed those of the general population, with approximately 60% of men and 3% of women living with HIV being smokers [4]. Text messaging is widely available in Vietnam [5,6], and text messaging interventions have been shown to be an effective strategy for improving smoking cessation outcomes [7-9].

Research among people living with HIV in the United States and other countries has identified several unique factors contributing to smoking, including co-occurring mental health challenges, substance use, HIV-related stigma, and limited access to cessation resources [10-13]. Community and family dynamics may also influence smoking behavior. In Vietnam, cigarette smoking is common for social bonding, especially in social situations such as bars and restaurants [14], whereas family dynamics may either motivate or deter cessation [14,15]. A systematic review found that smoking cessation interventions for people living with HIV are most effective when tailored to individual needs and include strategies to facilitate access to cessation resources [16]. Thus, understanding factors specific to the target population may provide additional insight into developing a text messaging intervention for people living with HIV in Vietnam.

Tobacco cessation efforts in Vietnam have focused on the establishment of tobacco cessation quitlines, telephone-based resources providing counseling support for individuals who smoke [17]. The Vietnam Ministry of Health established 2 quitlines (modeled after US quitlines) to support tobacco control, funded through the Tobacco Control Fund. Individuals who use quitlines can talk to staffed tobacco treatment specialists who are trained in tobacco cessation strategies as summarized in the 2008 US Treating Tobacco Use and Dependence guidelines [18]. However, these quitlines are underused [9,19]; only 2.7% of people who smoke and attempt to quit report receiving counseling [20]. Prior text messaging interventions have increased engagement with quitlines [9,21], suggesting that text messaging may provide an opportunity to increase quitline access among people living with HIV. For interventions focused on people living with HIV, these approaches may need to address specific factors that may act as barriers to using the quitlines.

During the intervention development phase of a randomized trial testing a text-based smoking cessation program for people living with HIV in Vietnam, we conducted a formative qualitative study to identify factors influencing smoking behaviors among people living with HIV in Vietnam. The qualitative study represented an initial step in the development of the text messaging intervention. In this paper, we report on our rigorous process for developing and conducting the qualitative interviews, identify key themes within preidentified domains, examine how these influences co-occur within everyday contexts, and describe how the findings informed the development of a guide for a subsequent peer-writing exercise in which people living with HIV who smoke were asked to write messages for others who were trying to quit smoking.

## Methods

### Study Design

This formative study was conducted as part of our M2Q2-HIV (mHealth Messaging to Motivate Quitline Use and Quitting among Persons Living with HIV) National Cancer Institute–funded clinical trial (NCT05863923). The overarching goal of the parent study was to develop and test a text messaging intervention [9] that incorporates peer messages (ie, prewritten messages from other individuals who smoke), tailored feedback for cessation goals, and quitline referrals for people living with HIV in Vietnam. Our qualitative study used a rapid qualitative analysis (RQA) approach, guided by methods described by Hamilton and Finley [22]. This approach is particularly useful in addressing the needs of marginalized communities through its action-oriented approach [23]. To enhance rigor, this study followed RQA recommendations from the Planning for and Assessing Rigor in Rapid Qualitative Analysis (PARRQA) framework [24]. This approach involved identifying key domains based on prior literature and expert feedback from team members [10-13,25], developing a semistructured interview guide, conducting qualitative interviews with 20 people living with HIV who smoke, and organizing and summarizing data within domains to support iterative analysis and theme development. The qualitative study was the first step in the development of our texting intervention, and the themes were used to develop the prompts for the guide used in the subsequent peer-writing exercise.

### Study Setting and Participant Recruitment

Participant inclusion criteria included a diagnosis of HIV infection (prevalent or incident HIV), age ≥18 years, and current smoking. Trial exclusion criteria included pregnancy or plans to become pregnant during the next 6 months, inability or unwillingness to provide informed consent, a prior diagnosis of a serious mental illness, and being a family member of another participant in the study. Patients were recruited from the same clinical settings in which the trial was conducted. Participants were recruited using convenience sampling [26] from 4 specialized HIV outpatient clinics that deliver antiretroviral therapy and other HIV-related treatment. These clinics were located in Hai Phong and Quang Ninh provinces in Vietnam. Participant recruitment began on September 14, 2022, and ended on September 24, 2022. Clinic staff and community health workers invited clinic patients to attend study screening events, where all patients who met the trial eligibility criteria were screened for study inclusion and exclusion by study staff. All eligible patients referred to the research team agreed to participate, with no documented refusals or reasons for nonparticipation. All participants completed informed consent procedures. Interviews were conducted on October 24, 2022, in Quang Ninh Province and on October 26, 2022, in Hai Phong Province.

### Interview Guide Development

The semistructured interview guide was developed and refined by research personnel, HIV experts in Vietnam, and the Vietnam quitline director. The interview guide was initially written in English and translated into Vietnamese by a certified translator. The interview guide addressed 6 different domains, including personal tobacco use, how and where participants observed tobacco use in their daily lives, how tobacco affects their mental and physical health, their experiences with HIV stigma in general and in relation to tobacco use, their experiences with tobacco cessation, and their experiences with alcohol and substance use.

### Data Collection

The interviews were conducted in person in a private setting within the participants’ HIV clinics. The interviews were conducted by trained research staff from the Institute of Population, Health and Development (PHAD), who used the semistructured interview guide. The interviews were audio-recorded and professionally transcribed. Interviewers were not involved in participants’ clinical care and had no prior relationship with participants. Interviews were initially transcribed in Vietnamese and then translated to English for analysis. Prior work has suggested that saturation occurs within 9 to 17 interviews, indicating that our sample size was sufficient for thematic depth [27].

### Qualitative Data Analysis

First, 2 members of the research team developed domains based on the interview guide and created a summary template organized around these initial thematic domains. This step was accomplished collaboratively with the larger study team, including the study’s principal investigator and coinvestigators, and refined through iterative discussion. Next, each transcript was randomly assigned to a primary analyst and a secondary analyst. Transcripts were independently reviewed and summarized by 1 analyst and then independently reviewed by the second analyst, ensuring that all transcripts were independently reviewed by 2 analysts. Analysts were instructed to read each transcript and record concise bullet-point summaries of participants’ observations and suggestions for each domain in the summary template. If no notable comments were made regarding a particular domain, analysts recorded “None” in the corresponding field. Team members met weekly to discuss independent analyses and resolve discrepancies through consensus. Transcript summaries were organized into a summary matrix to compare findings across participants and identify recurring patterns. The number of participants whose responses reflected each concept was recorded to provide context regarding the prevalence of specific perspectives. These findings were reviewed by the team and used to develop and refine the final themes. One member participated in these meetings as an independent reviewer and provided an additional perspective during discussions when clarification or adjudication was needed. The team also considered comments that diverged from emerging patterns and engaged in discussions about assumptions and interpretation when refining the final themes. An audit trail was maintained throughout the process to document changes to the summary template, matrix, and key analytic changes. All translated transcripts were reviewed by bilingual team members to verify accuracy and preserve meaning prior to analysis.

### Statistical Analysis

Descriptive statistics were used to summarize participant characteristics. Categorical variables were summarized using frequencies and percentages. Given the formative qualitative design of the study, no inferential statistical analyses were conducted.

### Ethical Considerations

This study was reviewed and approved by the institutional review board (IRB) at the PHAD in Vietnam (2022/PHAD/M2QHIV-01) and the University of Massachusetts Chan Medical School in Massachusetts (H00023608). All participants provided written informed consent prior to their inclusion in the study. Participant data were deidentified and maintained in accordance with procedures approved by the IRBs and the Declaration of Helsinki to protect participants’ privacy and confidentiality. Participants received an incentive of US $15 for participating in the interviews.

## Results

### Overview

The study consisted of 20 participants, 75% (n=15) of whom were aged 38 to 49 years and 25% (n=5) of whom were aged ≥50 years; 90% (n=18) were men. Half of the participants were from Quang Ninh Province, and the other half were from Hai Phong Province. Perceptions of overall tobacco use included an understanding of the harmful effects of tobacco but an inability to quit due to its addictive nature and habits formed at a young age. Among the 16 participants for whom smoking duration was reported, 11 (69%) reported smoking for at least 20 years, with some reporting that they started smoking as early as childhood or high school. The number of cigarettes smoked per day ranged from 7 cigarettes to 1 to 2 packs. The most popular brand of cigarettes was Song Cau, although many participants reported being willing to smoke any brand of cigarette. Among the 19 participants for whom prior quit attempts were reported, 13 (68%) had previously attempted to quit smoking, with some using chewing gum or candy as quitting strategies. All participants reported taking HIV medication, although the duration since HIV diagnosis was reported for only about 50% (n=10) of the sample. Current or previous alcohol use was reported by 15 (75%) participants, with a smaller number (>5 participants) reporting current or previous use of other substances such as drugs.

Analysis identified 6 themes within and across the preidentified domains, reflecting how social, psychological, and structural influences contributed to smoking behaviors.

### Theme 1: Social Contextual Factors Affect Smoking Behaviors

Individuals reported that they began smoking at a young age and were usually influenced to smoke by their friends or different groups with whom they were around at the time. Participants noted that smoking became a habit after they began smoking at a young age. One participant described their experience:

Generally, around the age of ten, I smoke, but I didn’t know when I became addicted, and then I started smoking again as a habit.

In adulthood, participants reported continuing to smoke due to established habits, seeing others smoke in public settings, and smoking at work. Participants described smoking because other people around them were smoking. One participant said:

We would be in a group of 5-6, drinking coffee and smoking cigarettes, or in a group of 9-10 people who will eat, drink and smoke.

On the other hand, participants also noted how social factors can motivate smoking cessation. Participants noted that their communities and interpersonal relationships influenced their motivation to quit smoking, for reasons such as wanting to prevent their family from experiencing the effects of smoking. One participant shared:

Smoking might help you feel relief now, but eventually, it will affect your wife, your children.

Similarly, another participant shared their belief that quitting smoking would improve their social relationships, stating:

It’s [quitting smoking] better in terms of the relationship with my friends and others.

### Theme 2: Smoking as a Coping Mechanism for Mental Health and Stress

Participants described the complex relationship between smoking and their mental health. They discussed how smoking sometimes provided stress relief, especially noting that their smoking increased during moments of stress and anxiety. One participant mentioned smoking when feeling down:

Sometimes, I smoke when I feel sad, or after a meal, my mouth feels empty, so I need to smoke cigarettes.

Others smoked to manage stress or anxiety:

I think normally, when I feel stressed, I immediately think of smoking cigarettes.

However, others reported that smoking caused mental health issues: “Smoking only 1-2 cigarettes per day makes me feel anxious and uncomfortable” and “makes my mental health weaker.” Smoking was also noted as a reason to quit to improve mental health, as 1 participant described smoking as making them “angry and uncomfortable...tobacco causes me a lot of discomfort.”

### Theme 3: Alcohol and Other Addictive Substances Alongside Cigarette Use Among People Living With HIV

Many participants described an increase in smoking while consuming alcohol, particularly in social settings:

It’s normal to smoke three cigarettes at a time. But if I drink alcohol, I will smoke up to ten cigarettes, and so will everyone. Smokers are often like that; if there are 100 people who drink alcohol, all of them will smoke.

Another participant noted the challenge of quitting due to alcohol and social influences:

I tried to quit a few times before but always relapsed when I’m out drinking coffee or beers with friends. When I’m alone, I hardly smoke any.

A participant also noted the impact of addictive substance use (including alcohol and tobacco) on self-control and behavior, stating:

When we use addictive substances, we cannot control our thoughts or actions, leading to inappropriate behavior.

### Theme 4: Living With HIV Shapes Smoking Behaviors and Motivations

Participants reported an increased level of smoking after an HIV diagnosis, attributing this difference to the distress associated with their diagnosis. For some participants, increased cigarette use served as a coping mechanism for dealing with societal stigma associated with HIV. One participant described this experience:

There is a difference. Before I didn’t know that I was infected with HIV, so I felt it was normal, but then I was infected with HIV, at that time HIV was not widely spread, so I was a bit shocked. So, I smoked tobacco a lot, even smoked two packs a day, just sat down and smoked, because of my bad mood. I could say that I smoke much more since I found out I got HIV.

Another participant shared a similar experience:

It does increase, for example, I smoked about 10 cigarettes, but after I knew I got HIV, the number of cigarettes increased, increasing to 5 more cigarettes.

Smoking appeared to also serve as a way to manage self-perception and emotional distress caused by HIV within social contexts. One participant explained:

Because as I feel self-conscious, I tend to smoke more, which annoys people around me. About my illness, sometimes it’s tired when using drugs, or I feel unease and uncomfortable with my body, so smoking cigarettes help me feel much better.

Although most participants noted a temporary increase in smoking after diagnosis, some participants noted that their smoking returned to normal over time.

### Theme 5: General Motivators for Quitting Centered on Financial and Health Benefits

Participants noted several general motivators that encouraged quitting, with financial and physical health concerns emerging as the most common. Several participants noted potential financial improvements as a result of smoking cessation. One participant shared:

Quitting smoking is great. The first reason is about my finances.

In contrast, another participant elaborated:

If I quit smoking now...it’s a reduction in my financial burden, as it’s costing me around hundreds of thousands VND [Vietnamese đồng].

Several participants mentioned the impact of smoking on their physical health. One participant reflected:

I think our lungs are like trees, protecting us from harmful dust, so, we must protect it.

Another noted:

Smoking makes me feel tired, and have phlegm, and as I get older, my health starts to worsen, so it’s good for me to quit smoking.

### Theme 6: Use of Quitlines, Texting, and Other Smoking Cessation Support

Participants mentioned a variety of reasons for wanting to quit, but other factors in their lives made it difficult to do so, particularly the lack of meaningful support. One participant said:

I think without any support; quitting can be hard.

Another participant noted that it would be helpful for support to go beyond just knowledge about the harmful effects of smoking:

I think health propaganda about the harmful effects of smoking is just like a reminder and most people ignore it. I don’t think a simple reminder would help.

Among existing cessation resources, the quitline was discussed by some participants, with 1 person noting a poor experience with the quitline:

I know a lot of cases where the operators tend to be annoyed or uncomfortable with us when we ask them questions.

Despite these challenges, participants offered insight into how to enhance cessation support. Some mentioned that the texting intervention could help with motivation:

I think if my phone could help remind me whenever I crave, then it would help and motivate me more.

Another participant emphasized this point:

I think that if the project could have someone that encourages and motivates us to quit, then it would be best. Because the best method is to have someone to help change your behavior. Now, I don’t have a lot of motivation, but if the project could help me with this, then it would be the best.

Across these themes, participants described smoking as part of their everyday lives, shaped by social situations, stress, alcohol use, and their experiences living with HIV. Although many identified financial and health reasons to quit, quitting remained difficult without consistent and meaningful support. Smoking was often described as occurring in response to multiple challenges rather than as a single, isolated behavior.

Table 1 lists how these themes informed the guide for the peer message writing exercise. It also provides examples of messages that were used in the intervention to address these themes. The full peer message writing guide in English is provided in Multimedia Appendix 1, and the Vietnamese translation of the intervention messages is provided in Multimedia Appendix 2.

**Table 1.** Qualitative themes, peer message writing prompts, and example messages for smoking cessation among people living with HIV in Vietnam: M2Q2-HIV (mHealth Messaging to Motivate Quitline Use and Quitting among Persons Living with HIV) Study (2022-2023).

Qualitative themes	Key participant insight	Prompt examples	Message examples
Social contextual factors affecting smoking behaviors	Participants described the influence of family and friends, noting the habitual nature of smoking. Smoking occurred in many public settings, including the workplace. Participants also noted how their family and social relationships may benefit from quitting smoking.	“I asked the people around me to help quit smoking by...” “People often smoke when socializing with friends. How can someone reduce the temptation to smoke around their friends.”	“If you don’t really want to stop smoking yet, remember it is important that smoking directly affects your health, loved ones and family.”
Smoking as a coping mechanism for mental health and stress	Participants noted that smoking provided stress relief, especially during moments of anxiety. Other participants noted negative mental health effects from smoking.	“When preparing to stop smoking, one of the most important things someone can do for their mental health is:”	“One important reason someone with a mental health issue may want to stop smoking is that tobacco hasn’t the advantage to reduce stress, and anxiety, on the contrary, it makes you more tired and bored.”
Alcohol and other addictive substances alongside cigarette use among people living with HIV	Participants noted the addictive nature of other substances and how this may influence them to smoke more, even when they are trying to quit.	“When tempting situations arise, it is often hard for people to continue with their quit attempt. What can someone do to stay focused on quitting?”	"I have asked people around me to help me quit smoking by not inviting me to smoke, limiting or not hanging out in sidewalk bars.”
Living with HIV shapes smoking behaviors and motivations	Participants noted that the nature of their HIV diagnosis led to a significant increase in smoking, including using cigarettes to manage stress resulting from their HIV diagnosis.	“When I felt down about my HIV diagnosis, instead of smoking, I sought support from...”	“When I felt down about my HIV diagnosis, instead of smoking, I sought support from my family, my friends, and the people around me to stop thinking too much and feel sociable with everyone.”
General motivators for quitting centered on financial and health benefits	Participants noted that health and financial benefits could pose as motivators for quitting.	“I quit smoking because...”	“I quit smoking because tobacco is very harmful to my health and my financial...”
Use of quitlines, texting, and other smoking cessation support	Participants noted that smoking support is usually not tailored to their specific needs but also noted that having a phone and receiving frequent messages may increase motivation to quit.	“Some people seek help when quitting smoking from doctors at clinics or quitlines. What resources would you recommend to someone to use on their quit day?”	“Don’t forget to search online, visit counseling centers and ask people around you for resources and motivation to quit tobacco.”

## Discussion

### Principal Findings

We identified how social context, mental health, alcohol and substance use, and HIV-related experiences shape smoking behaviors. Across these influences, smoking often functioned as a socially reinforced coping strategy rather than as the result of isolated factors. We also identified how general motivations and perceptions of cessation support influence participants’ willingness and ability to quit smoking. Overall, these accounts suggest that smoking functioned as a coping strategy and socially reinforced behavior and that smoking among people living with HIV in Vietnam is embedded within intersecting contexts.

Participants’ accounts suggest that social settings normalized smoking, but at the same time, concern for family members and relationships emerged as a social influence that could motivate quitting, indicating that social context played both reinforcing and protective roles. Previous studies have focused on interpersonal relationships and household bans on smoking in the home environment to encourage cessation and protect family members [28]. There is great importance in addressing the social contexts of smoking, particularly in settings where smoking is culturally normalized [29]. Our findings underscore the broader impact of public spaces and workplaces, as attitudes toward smoking and actual cessation behaviors in Vietnam vary. Interventions that focus on reshaping social norms using peer messaging, such as our planned texting intervention, could promote smoking cessation among people living with HIV in Vietnam, but this will need to be tested in a randomized trial such as our planned study.

The mental health effects of smoking also had a major influence on participants, as some participants noted that smoking helped them cope. Smoking and mental health are closely linked; many people use smoking as a coping behavior [30,31]. However, many studies show that smoking can exacerbate mental health symptoms [32,33], making quitting smoking more difficult [34]. Studies in Vietnam also show this interdependence, as 1 study estimated that depressive symptoms have been reported as being 16 times higher in people living with HIV who smoke than in the general Vietnamese population with higher tobacco dependence [1], and another study found that people living with HIV with higher tobacco dependence reported more depressive symptoms [35]. Many of our participants also reported that using smoking as a coping strategy for mental health symptoms often occurred in social contexts. These descriptions indicate that stress and social interaction were closely linked in participants’ experiences of smoking. Similarly, many participants discussed how alcohol use and smoking co-occurred in group settings and during stressful situations. Despite this, some participants in our study reported that smoking helped their mental health, with some using it as a coping mechanism. Interventions should consider how to incorporate stress-relief support to replace smoking.

Many participants discussed their HIV diagnosis as a factor in their smoking. Participants noted large increases in their smoking, with some increasing their smoking by 2- or 3-fold after receiving their HIV diagnosis. Smokers with HIV typically report higher rates of smoking, with the prevalence of smoking among people living with HIV being approximately 1.3-fold that of the general population [36]. Few participants mentioned any facilitators to quitting behaviors as a result of their HIV diagnosis, suggesting that HIV-related care settings might be missing an opportunity to provide smoking cessation support [15,37]. This also highlights the need for approaches that acknowledge HIV-related experiences when supporting smoking cessation.

Quitlines have been shown to support smoking cessation, with some quitlines increasing cessation rates from 7% to 10% [38]. Quitline studies in Vietnam have reported that 5.1% of participants stopped smoking completely at the 6-month follow-up [19]. However, despite the quitline’s moderate success, our study suggests that people living with HIV in Vietnam are mostly unaware of quitline support. In a previous study, a quitline in Vietnam was shown to have received only 25 calls per day, representing only a fraction of smokers in that region [9]. Prior research has also shown that two-thirds of people living with HIV who smoke did not want to connect with the quitline [39], with others reporting that between 50% and 80% of people living with HIV who smoke were uninterested in quitting or cessation programs [40]. Studies have also shown that using texting interventions to increase the reach of and knowledge about quitline services can be successful [18,41]. In our previous work testing the M2Q2-HIV intervention in Vietnam among smokers without HIV, text messaging substantially increased verified quitline use [9], while other work in the United States has also shown similar cessation rates among people living with HIV and people without HIV who smoke when participating in the quitline [39]. However, participants in our study who used the quitline indicated that the support they received was less than optimal. These findings may reflect a broader gap between HIV clinical care and existing smoking cessation resources in Vietnam. Vietnam has invested in quitline services, but HIV clinics and quitline services operate separately. Strengthening connections between clinics and existing resources may highlight a need for increasing access to cessation support that addresses HIV-related stigma and the unique needs of people living with HIV.

Views on texting interventions were mixed. Some participants described the texting intervention as a potential source of motivation to quit, particularly given their frequent phone use and the possibility of receiving messages from other people living with HIV who smoke. However, some participants reported that the messages could be annoying or unhelpful. A prior text messaging intervention study demonstrated increased confidence in quitting and smoking abstinence by the end of treatment [8,42,43]. Studies among people living with HIV in other countries have reported high engagement with texting interventions and suggest potential for efficacious implementation in similar settings [44,45].

Overall, these findings indicate that smoking among people living with HIV in Vietnam was shaped by overlapping social environments, emotional distress, alcohol use, and HIV-related experiences. Rather than acting independently, these influences often occurred together, reinforcing smoking within daily routines and interpersonal settings. Therefore, interventions that address only 1 factor in isolation may be insufficient. In the subsequent peer message writing exercise conducted as part of our project to develop the text messaging intervention, participants responded to prompts developed across the identified themes, including several themes that addressed overlapping influences on smoking. As illustrated in Table 1, the resulting messages reflected the multiple factors that participants described as contributing to smoking and cessation. Our overall project is evaluating these messages in a randomized trial among people living with HIV in Vietnam.

### Limitations

Our study had several limitations. Participants self-reported their tobacco use. Our sample was made up of 90% (n=18) men, which, although reflective of the composition of people living with HIV who smoke in Vietnam (only 3% of women smoke) [4], could limit the generalizability of our results. Coding was performed on English translations rather than the original Vietnamese transcripts. Although the analytic team included Vietnamese-speaking members who could consult the original transcripts, most coders worked from the translations. Thus, some subtle cultural idioms, colloquial phrasing, and emotional nuances regarding HIV stigma and mental health may have been missed. Additionally, our study focused specifically on patients receiving treatment at HIV clinics in Hai Phong and Quang Ninh provinces, limiting our findings to patients seeking treatment at these clinics.

### Conclusions

People living with HIV in Vietnam experience unique life stressors as a result of their HIV diagnosis and a lack of personalized cessation tools. Their smoking behaviors are impacted by these conditions, which, if unaddressed, can make quitting more difficult. These findings informed the development of a text messaging intervention tailored to the experiences of people living with HIV who smoke. These findings may also be relevant in other settings where people living with HIV who smoke are underserved.

## Supplementary material

10.2196/97427 Multimedia Appendix 1 Supplemental message writing activity workbook in English.

10.2196/97427 Multimedia Appendix 2 Supplemental message writing activity workbook in Vietnamese.

## Abbreviations

IRB: institutional review board
M2Q2-HIV: mHealth Messaging to Motivate Quitline Use and Quitting among Persons Living with HIV
PARRQA: Planning for and Assessing Rigor in Rapid Qualitative Analysis
PHAD: Institute of Population, Health and Development
RQA: rapid qualitative analysis
