# Supplementary material for: Understanding the Influence of Substance Use, Mental Health, and Stigma on Smoking Behavior Among People Living With HIV: Rapid Qualitative Analysis
Source: JMIR Form Res. 2026 Oct 15;10:e97427. doi: 10.2196/97427 (PMC13671598; doi:10.2196/97427)
Supplement: Supplementary file 1 [file formative-v10-e97427-s001.pdf]

Message Writer ID___________________

M2Q2-HIV

mHealth Messaging to Motivate Quitline use and Quitting among Persons Living With HIV in Vietnam

TEXT MESSAGE WRITING ACTIVITY

WORKBOOK

Exercise 1:

Sometimes it is difficult to decide to quit smoking. What made you quit?

a. *Message Prompt:* I quit smoking because____.

Exercise 2:

People around you can play a big role in quitting smoking. Think about things your family and friends did to help you stop smoking.

a. *Message Prompt*: I asked the people around me to help me quit smoking by ____.

b. *Message Prompt* : If being around people who smoked made it more difficult for me to quit, I would ____.

c. *Message prompt*: People often smoke when socializing with friends. How can someone reduce the temptation to smoke around their friends? Please give examples:
   a.____.
   b.____.
   c. ____.

Exercise 3:

Think about how you perceive yourself since your HIV diagnosis, and how others perceive and treat you. What would you tell someone who has HIV and smokes, but wants to quit?

a. *Message Prompt:* Being judged for my HIV status made me want to smoke – when this happened, I found it helpful to____.

b. *Message Prompt:* When I felt down about my HIV diagnosis, instead of smoking, I sought support from____.

c. *Message Prompt:* ____really helped me manage the stress of my HIV diagnosis without smoking.

Exercise 4:

Some people living with HIV who smoke have mental health conditions and may use smoking to help manage their symptoms. What might you tell someone living with HIV and a mental health condition who smokes about quitting smoking?

a. *Message prompt:* What are two important things that you would tell someone with a mental health condition who is **not** ready to stop smoking?
   1.____.
   2.____.

b. *Message prompt:* One important reason someone with a mental health issue may want to stop smoking is:
   ____.

c. *Message prompt:* One immediate change in mental health someone may notice after they stop smoking is:____.

d. *Message prompt:* For people with mental health conditions, what are some of the negative impacts smoking might have on their lives? Please give examples:
   a.____.
   b.____.
   c. ____.

e. *Message prompt:* When preparing to stop smoking, one of the most important things someone can do for their mental health is: ____.

Exercise 5:

Quitting smoking can be hard. Think about the difficulties you had when you tried to stop smoking. These could include family interactions while quitting, changes in your mood, and experiencing cigarette cravings.

a. *Message prompt:* Imagine someone who has stopped smoking for a week but has started experiencing cigarette cravings. What would you suggest to reduce cigarette cravings? Please give examples:
   a.____.
   b.____.
   c. ____.

b. *Message prompt:* People often smoke when bored. What activities, other than smoking, would you suggest doing when bored? Please give examples:
   a.____.
   b.____.
   c. ____.

c. *Message prompt:* How would you encourage someone to stay motivated to continue being cigarette-free? Please give examples:
   a.____.
   b.____.
   c. ____.

d. *Message prompt:* When people initially stop smoking, they tend to experience low moods. What advice would you give to overcome this challenge? Please give examples:
   a. ____.
   b. ____.
   c. ____.

e. *Message prompt:* Some people seek help when quitting smoking from doctors at clinics or quitlines. What resources would you recommend to someone to use on their quit day? Please give examples:
   a. ____.
   b. ____.
   c. ____.

f. *Message prompt:* When tempting situations arise, it is often hard for people to continue with their quit attempt. What can someone do to stay focused on quitting? Please give examples:
   a. ____.
   b. ____.
   c. ____.
