# Supplementary material for: Understanding the Influence of Substance Use, Mental Health, and Stigma on Smoking Behavior Among People Living With HIV: Rapid Qualitative Analysis
Source: JMIR Form Res. 2026 Oct 15;10:e97427. doi: 10.2196/97427 (PMC13671598; doi:10.2196/97427)
Supplement: Supplementary file 2 [file formative-v10-e97427-s002.pdf]

ID người viết tin nhắn ____________________

M2Q2-HIV

Ứng dụng hệ thống tin nhắn (mHealth) để thúc đẩy sử dụng tổng đài tư vấn cai nghiện thuốc lá Quitline và cai thuốc lá trên những người nhiễm HIV tại Việt Nam

HOẠT ĐỘNG VIẾT TIN NHẮN

TÌNH HUỐNG VÀ MỘT SỐ GỢI Ý

ID người viết tin nhắn ____________________

Tình huống 1:

Đôi khi thật khó để quyết định cai thuốc lá. Điều gì khiến anh/chị muốn cai thuốc lá?

a. *Gợi ý tin nhắ*n: Tôi cai thuốc lá bởi vì____.

Tình huống 2:

Mọi người xung quanh anh/chị có thể đóng một vai trò lớn trong việc cai thuốc lá của anh/chị. Hãy nghĩ về những điều gia đình hoặc bạn bè của anh/chị đã làm để giúp anh/chị cai hút thuốc lá.

a. *Gợi ý tin nhắn:* Tôi đã yêu cầu mọi người xung quanh mình giúp tôi cai thuốc lá bằng cách ____.
b. *Gợi ý tin nhắn:* Nếu ở gần những người hút thuốc lá khiến tôi khó cai thuốc lá hơn, tôi sẽ ____.
c. *Gợi ý tin nhắn:* Mọi người thỉnh thoảng hút thuốc lá khi xã giao cùng bạn bè. Làm như thế nào để một người có thể giảm bớt cám rỗ hút thuốc lá xung quanh bạn bè của họ. Hãy cho một vài ví dụ:
    a) ____.
    b) ____.
    c) ____.

Tình huống 3:

Anh/chị nhận thức như thế nào về bản thân từ khi anh/chị bị chẩn đoán nhiễm HIV. Và những người khác nhận thức và đối xử với anh chị như thế nào? Anh/chị sẽ nói gì với một người nhiễm HIV đang hút thuốc lá nhưng muốn cai thuốc lá?

a. *Gợi ý tin nhắn:* Bị đánh giá vì nhiễm HIV khiến tôi muốn hút thuốc lá – khi đó, tôi cảm thấy thuốc lá hữu ích để____.
b. *Gợi ý tin nhắn:* Khi tôi chán nản, thất vọng vì chẩn đoán nhiễm HIV của mình, thay vì hút thuốc lá, tôi tìm kiếm sự giúp đỡ từ____.
c. *Gợi ý tin nhắn:* ____thực sự đã giúp tôi kiểm soát căng thẳng vì chẩn đoán HIV của mình, mà không cần dùng đến thuốc lá.

Tình huống 4:

Một số người nhiễm HIV hút thuốc lá có những vấn đề sức khỏe tâm thần và có lẽ việc sử dụng thuốc lá giúp họ kiểm soát những triệu chứng của mình. Anh/chị có thể nói điều gì với **một người như vậy** về việc cai thuốc lá?

ID người viết tin nhắn ____________________

a. *Gợi ý lời nhắn:* Hai điều quan trọng nào mà anh/chị có thể nói với **người đó**, mặc dù **người đó** chưa thực sự muốn dừng hút thuốc lá?

1. ____.
2. ____.

b. *Gợi ý tin nhắn:* Một lý do quan trọng đối với một người có vấn đề về sức khỏe tâm thần muốn dừng việc hút thuốc lá là:

____.

c. *Gợi ý tin nhắn:* Một thay đổi ngay lập tức ở sức khỏe tâm thần mà một người có thể nhận thấy sau khi người đó dừng hút thuốc lá là:______________________________________.

d. *Gợi ý tin nhắn:* Với những người có vấn đề về sức khỏe tâm thần, những tác động tiêu cực của việc hút thuốc lá có thể có đối với cuộc sống của họ là gì? Hãy cho một vài ví dụ:

a) ____.
b) ____.
c) ____.

e. *Gợi ý tin nhắn:* Khi chuẩn bị ngừng hút thuốc lá, một trong những điều quan trọng nhất một người có thể làm cho sức khỏe tâm thần của mình là:

________________________________.

Tình huống 5:

Cai được thuốc lá là một việc khó khăn. Hãy nghĩ về những khó khăn anh/chị gặp phải khi anh/chị cố gắng để ngừng hút thuốc lá. Có thể bao gồm tương tác của gia đình trong khi bỏ thuốc, những thay đổi cảm xúc của anh/chị, và trải qua những cơn thèm thuốc lá.

a. *Gợi ý tin nhắn:* Tưởng tượng ai đó đã dừng hút thuốc lá được 1 tuần, nhưng người đó lại bắt đầu trải qua những cơn thèm thuốc. Anh/chị có thể khuyên người đó điều gì để giảm bớt những cơn thèm thuốc lá của họ? Hãy cho một vài ví dụ:

a) ____.
b) ____.
c) ____.

b. *Gợi ý tin nhắn:* Mọi người thường hút thuốc lá mỗi khi buồn chán. Vậy có những hoạt động nào (khác hút thuốc lá) mà anh/chị có thể gợi ý làm mỗi khi buồn chán? Hãy cho một vài ví dụ:

a) ____.
b) ____.
c) ____.

c. *Gợi ý tin nhắn:* Làm cách nào anh/chị cổ vũ ai đó duy trì động lực để tiếp tục không dùng thuốc lá? Hãy cho một vài ví dụ:

a) ____.
b) ____.
c) ____.

d. *Gợi ý tin nhắn:* Ban đầu khi mọi người dừng hút thuốc lá, trạng thái tâm lý của họ đi xuống. Anh/chị có lời khuyên nào để vượt qua thử thách này không? Hãy cho một vài ví dụ:

a) ____.

b) ____.
c) ____.

e. *Gợi ý tin nhắn:* Một số người tìm kiếm sự giúp đỡ khi cai thuốc lá từ bác sỹ ở phòng khám hoặc tổng đài cai nghiện thuốc lá (Quitline). Anh/chị có đề xuất những nguồn – tài liệu để ai đó sử dụng trong những ngày cai thuốc lá của người đó không? Hãy cho một vài ví dụ:

a) ____.
b) ____.
c) ____.

f. Gợi ý tin nhắn: Khi tình huống hấp dẫn phát sinh, mọi người thường khó tiếp tục nỗ lực bỏ thuốc lá của mình. Ai đó có thể làm gì để tập trung vào việc bỏ thuốc lá? Hãy cho một vài ví dụ:

a) ____.
b) ____.
c) ____.
